# Characteristics of Cancer Epidemiology Studies That Employ Metabolomics: A Scoping Review

**DOI:** 10.1158/1055-9965.EPI-23-0045

**Published:** 2023-07-06

**Authors:** Catherine T. Yu, Zeinab Farhat, Alicia A. Livinski, Erikka Loftfield, Krista A. Zanetti

**Affiliations:** 1 Epidemiology and Genomics Research Program, Division of Cancer Control and Population Sciences, National Cancer Institute, Rockville, Maryland.; 2 Metabolic Epidemiology Branch, Division of Cancer Epidemiology and Genetics, National Cancer Institute, Rockville, Maryland.; 3 National Institutes of Health Library, Office of Research Services, Office of the Director, National Institutes of Health, Bethesda, Maryland.; 4 Office of Nutrition Research, Division of Program Coordination, Planning, and Strategic Initiatives, Office of the Director, National Institutes of Health, Bethesda, Maryland.

## Abstract

An increasing number of cancer epidemiology studies use metabolomics assays. This scoping review characterizes trends in the literature in terms of study design, population characteristics, and metabolomics approaches and identifies opportunities for future growth and improvement. We searched PubMed/MEDLINE, Embase, Scopus, and Web of Science: Core Collection databases and included research articles that used metabolomics to primarily study cancer, contained a minimum of 100 cases in each main analysis stratum, used an epidemiologic study design, and were published in English from 1998 to June 2021. A total of 2,048 articles were screened, of which 314 full texts were further assessed resulting in 77 included articles. The most well-studied cancers were colorectal (19.5%), prostate (19.5%), and breast (19.5%). Most studies used a nested case–control design to estimate associations between individual metabolites and cancer risk and a liquid chromatography–tandem mass spectrometry untargeted or semi-targeted approach to measure metabolites in blood. Studies were geographically diverse, including countries in Asia, Europe, and North America; 27.3% of studies reported on participant race, the majority reporting White participants. Most studies (70.2%) included fewer than 300 cancer cases in their main analysis. This scoping review identified key areas for improvement, including needs for standardized race and ethnicity reporting, more diverse study populations, and larger studies.

## Introduction

Metabolomics is an “omics” approach focused on the large-scale analysis of the metabolome, the set of metabolites within a biological system (1, 2). The emergence of the field can be traced to 1998, when the term “metabolome” was first introduced by Oliver and colleagues (3). Metabolomics has been shown to be a powerful tool for studying human health and biology. It can be applied to estimate disease risk, elucidate biological mechanisms, and identify biomarkers for disease diagnosis and prognosis. Two main analytic approaches are used in these studies: untargeted and semi-targeted profiling. Untargeted studies aim to detect as many metabolites as possible using a global approach, where there is no *a priori* metabolite information leading to data acquisition (4). Semi-targeted studies profile hundreds of metabolites whose identity is defined from a range of chemical classes and metabolic pathways before experimentation (4). Additionally, there are targeted analyses, which quantify a smaller number of predefined metabolites that are related in function and class (4). To detect metabolites in a sample, commonly used metabolomic platforms include mass spectrometry (MS)–based and nuclear magnetic resonance (NMR)–based techniques. MS-based platforms have the advantages of broader metabolite coverage and higher sensitivity compared with NMR, but they are destructive to the sample, and technical reproducibility is variable (5). In contrast, NMR-based platforms provide detailed structural information on fewer metabolites and are nondestructive and fully quantitative (5).

There has been a significant investment into the field of metabolomics from the National Institutes of Health (NIH), which has catalyzed its use in epidemiology to study human diseases, including cancer (6). In 2012, the NIH Common Fund Metabolomics Program was established to address key challenges in the field, including the need for improved rigor and reproducibility, and establish an infrastructure for metabolomics in the United States (7). Following this initial investment, the international COnsortium of METabolomics Studies (COMETS) was established in 2014 to develop methods to harmonize data, develop data analysis tools specific to these studies, and build a network of epidemiologists in the field (6, 8, 9).

Metabolomic epidemiology, as defined by Lasky-Su and colleagues (10) is “the field of scientific enquiry involving the systematic use of epidemiological methods and principles to study population-based variation in the human metabolome as it associates with health-related outcomes or exposures.” Case–control, cross-sectional, prospective cohort, and nested study designs are common types of metabolomic epidemiology studies (10–12). Case–control and cross-sectional studies allow researchers to glean potential metabolomic differences by comparing individuals by disease or exposure status (12). Case–control studies also involve biospecimen collection at the time of diagnosis, allowing for stronger metabolite–disease associations (11). Studies that use prospective sampling allow the assessment of temporal relationships (13). Biospecimens that are typically collected in epidemiologic studies are suitable for metabolomics analysis (10, 14). However, metabolites are known to be affected by preanalytical conditions such as biospecimen collection, processing, and storage conditions; therefore, experimental processes should be consistently applied across all biospecimens (15).

To date, comprehensive reviews in the field of metabolomic epidemiology are lacking. Therefore, we launched a scoping review to systematically map the field of population-based cancer metabolomics research, characterizing trends in the literature in terms of study design, population characteristics, and metabolomics approaches, and to identify opportunities for future growth and improvement.

## Materials and Methods

### Protocol and registration

We used the Preferred Reporting Items for Systematic Reviews and Meta-Analyses (PRISMA) extension for Scoping Reviews to write the protocol and final reporting of this review (16). The Materials and Methods section outlines the methodological protocol used for the study.

### Eligibility criteria

We used the following inclusion criteria for the study selection process: (i) the article used metabolomics to study cancer; (ii) cancer was the focus of the article; (iii) the article contained at least 100 cases in each analysis stratification of the main analysis; (iv) the article used an epidemiologic study design; and (v) and the article was published in English from 1998 to June 2021. We also included studies based in an international setting to capture studies investigating international cohorts.

For this review, our definition of metabolomics included untargeted and semi-targeted metabolic profiling, assessment of two or more Kyoto Encyclopedia of Genes and Genomes (KEGG) pathways (17), and lipidomics. Although lipids are represented under a single KEGG pathway, we included lipidomics in our definition due to the large number and wide diversity of metabolites in the biochemical class. We excluded from our definition of metabolomics targeted analyses. Articles must have had a minimum of 100 cases in each analysis stratification of the main analysis to meet our definition of a population-based epidemiology study (2). If the study included training and validation sets, then the discovery set was considered the main analysis. We limited to the English language only as our review team did not have translation capabilities available, and publication year 1998 because the scientific term “metabolome” was first introduced in 1998 (3).

### Information sources and search

Search strategies were developed using keywords and controlled vocabularies (i.e., MeSH, Emtree) for metabolomics, cancer, and epidemiologic study designs and four databases were searched by a biomedical librarian (AAL): PubMed/MEDLINE (United States National Library of Medicine), Embase (Elsevier), Scopus (Elsevier), and Web of Science: Core Collection (Clarivate Analytics). All searches were conducted in April and June 2021. The searches were limited by publication date (January 1, 1998–June 1, 2021) and language (English), and animal studies and specific article types not reporting data (e.g., retractions, corrigenda, errata, reviews, editorials, commentaries, letters, conference abstracts/proceedings, and meta-analyses) were excluded. A search strategy was used to exclude animal studies and article types. All database results were exported to EndNote X9.3.3 (Clarivate Analytics), and the find duplicates feature was used to identify unique articles.

### Selection of sources of evidence

We performed a pilot of the study selection process (both levels) with four authors (ZF, EL, CTY, and KAZ) on a random sample of records in Covidence (Veritas Health Innovation) and the eligibility criteria were revised as necessary.

All unique records were imported by AAL in XML format into Covidence for study screening. Each article at the title and abstract and full-text level was screened independently by two authors. First, three authors (ZF, CTY, and KAZ) independently screened the titles and abstracts of all unique records using the predefined eligibility criteria. Next, the same three authors independently screened the full text of those articles included after title and abstract screening against our eligibility criteria. For both levels of study selection, EL resolved disagreements between authors during the screening through an independent review.

### Data charting process and data items

Data charting was performed in Qualtrics (Qualtrics XM) using a form that was tested with all participating authors prior to its use. Three authors (ZF, CTY, and KAZ) performed the data charting, and each article was charted independently by two authors. The list of charted data items, including any assumptions and definitions used, is described below. Following data charting, SAS 9.4 (SAS institute) was used to identify discrepancies in the extracted data. Discordances in the data collected were resolved by EL and through group discussion when necessary.

We extracted the following data items from each article, when available:

- Publication year
- Author name
- Epidemiologic study design
- Cancer type
- Population characteristics (i.e., cohort name, sample size, age range of study participants, and participant race and ethnicity)
- Study setting (i.e., country(ies) from where the study population was chosen)
- Primary metabolomics-specific research aim
- Analytic platform used to perform metabolomics
- Metabolomics approach (i.e., untargeted or semi-targeted)
- Biospecimen type
- Whether the study compared race/ethnic groups in some way

All data items were presented as closed-ended questions with prespecified responses, except for author name, publication year, cohort name, and sample size. When authors selected “other” during extraction, they were required to provide an additional brief explanation of the data item. When authors selected “yes” for whether a study performed comparisons by race or ethnicity, they were required to provide a brief explanation of the comparisons and relevant findings. Data items that were not available in the article were recorded as missing.

We categorized epidemiologic study designs as follows, based on standard epidemiology definitions (18,19):

- Case–control study
- Case–cohort study
- Case-only study
- Case-series study
- Cohort study
- Cross-sectional study
- Intervention trial
- Nested case–control study
- Other: Any study design not fitting into the categories

Studies that did not investigate a pediatric cancer or specify participant age range but did report a mean age >18 years were categorized as adult studies. We used the five race categories outlined by the United States Office of Management and Budget (OMB) for our collection of study population race data: American Indian or Alaska Native, Asian, Black or African American, Native Hawaiian or Other Pacific Islander, and White (20). When studies reported race data that did not fit the OMB-outlined categories, the authors recorded the race data as “other.’’ For extraction of study population ethnicity data, we used the categories "Hispanic or Latino" and "Not Hispanic or Latino,” also as outlined by OMB standards (20). The authors made no assumptions about the race and ethnicity of study participants and recorded race and ethnicity data items as directly communicated by the paper. Following data extraction and discordance resolution, CTY reviewed the articles recorded as reporting race data to determine if the papers reported disaggregated subgroup information. ZF and CTY also charted cancer case, diseased control, and healthy control numbers *post hoc*. KAZ resolved discordances between ZF and CTY.

We used the following prespecified research aim categories when extracting data about an article's primary objective for using metabolomics:

- Cancer risk estimation using incident cases
- Cancer risk estimation using prevalent cases
- Risk of recurrence or death among cancer survivors
- Treatment intervention with an outcome of cancer
- Lifestyle intervention with an outcome of cancer
- Cancer progression/natural history
- Biomarker of exposure and cancer risk estimation using incident cases
- Biomarker of exposure and cancer risk estimation using prevalent cases
- Other.

We defined “cancer risk estimation using prevalent cases” as studies that estimated associations of metabolites with cancer prevalence. Studies that were recorded as “other” were further reviewed following data charting by CTY and KAZ to identify additional research aims categories. The additional aim categories included biomarkers of disease diagnosis, biomarkers of survival, biomarkers of disease diagnosis and survival, disease differentiation: cases vs. controls (compares cases vs. controls to examine whether disease is present), disease differentiation: tumor vs. non-tumor (compares tumor vs. non-tumor tissue to determine if disease is present), association study: prognosis/recurrence, and descriptive study: progression/survival. To be considered a biomarker study, the analysis needed to include a receiver operating characteristic curve, otherwise the study was considered either as association or descriptive.

We categorized the metabolomics approaches used as:

- Untargeted: Metabolomics study applied for wide detection of metabolites in a sample, ranging from 100s to 1,000s of metabolites (4). These studies lack knowledge of its metabolite targets prior to experimentation and are not quantitative (4).
- Semi-targeted: Metabolomics study applied to profile 100s of metabolites in a sample (4). These studies use predefined metabolite targets that are chosen from several metabolic pathways and chemical classes of biological interest which cover a wide range of metabolism (4).

Following data extraction and discordance resolution, ZF and CTY independently reviewed the articles recorded as performing semi-targeted metabolomics to further extract the metabolites targeted in these studies. CTY then categorized the metabolites according to the eight super-pathways profiled and defined by Metabolon, Inc.: amino acids, carbohydrates, cofactors and vitamins, energy, lipids, nucleotides, peptides, and xenobiotics (21, 22). Metabolites that did not fall under a Metabolon super-pathway were categorized as “other.”

#### Synthesis of results

We collected descriptive statistics from the included studies and summarized in a narrative format, as well as reported visually using pie charts, bar graphs, and map formats the charted data items. CTY performed analyses in RStudio Version 1.3.1093 (RStudio) and Excel Version 2108 (Microsoft). All figures were created using Excel Version 2108 (Microsoft).

## Results

### Selection of sources of evidence

**Figure 1.**
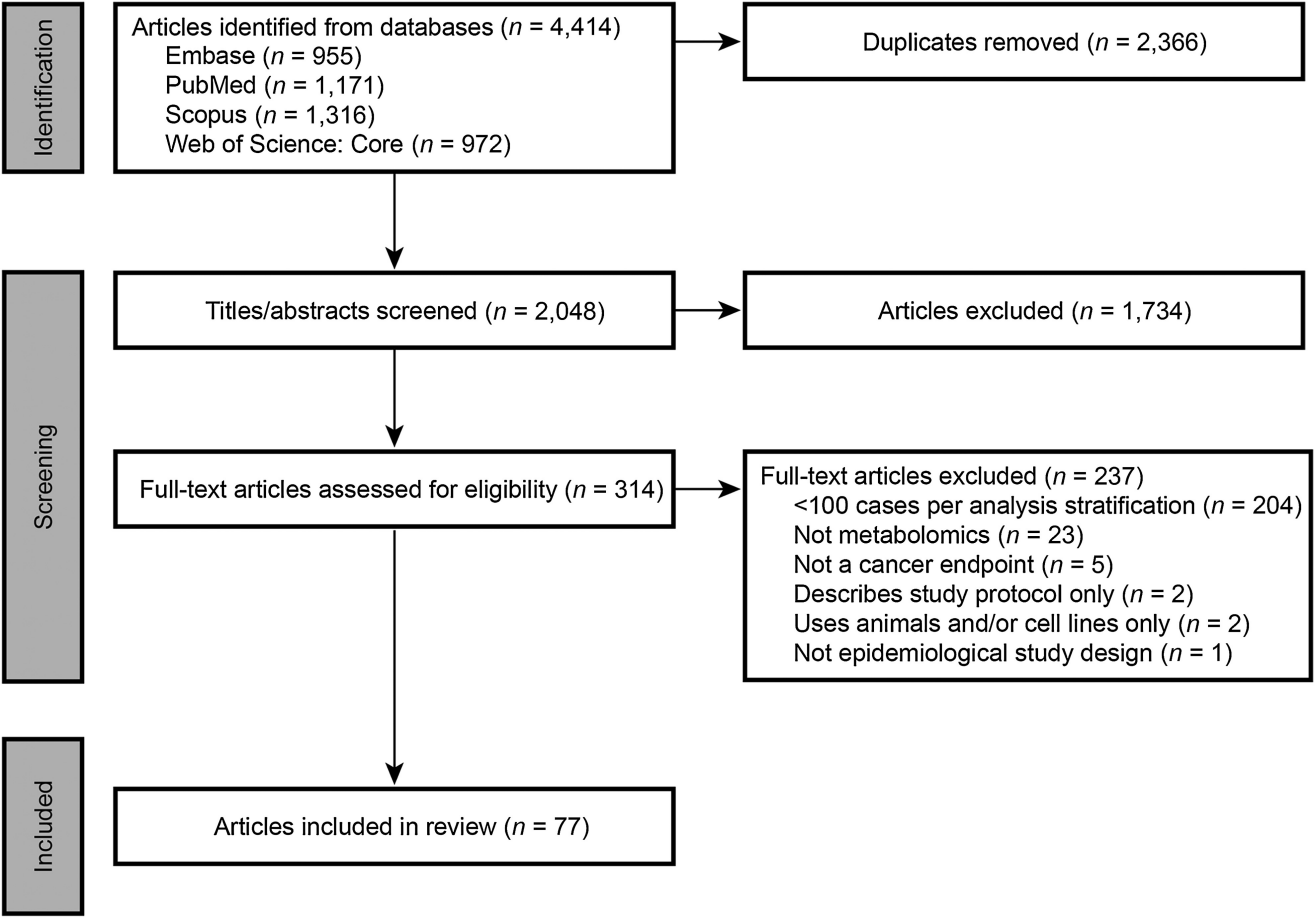
PRISMA flow diagram for selecting sources of evidence. The flow diagram shows the process used to select sources of evidence to be included in the scoping review examining population-based cancer metabolomics research.

The database searches identified 4,414 articles and 2,366 duplicates were removed prior to screening. The titles and abstracts of 2,048 articles were screened, of which 1,734 were excluded. The full text of 314 articles was then assessed for eligibility, of which 237 were excluded and 77 articles were included in the review. Figure 1 displays the flow of information throughout the review and the reasons for exclusion at full-text screening.

### Key characteristics of included studies

For each study included in this review, Table 1 presents the key characteristics and data collected including study design, cancer type, study setting, sample size, age range, race and Hispanic ethnicity groups reported, metabolomics-specific research aim, metabolomics approach, metabolomics analytic platform, and biospecimen.

**Table 1.** Study-, population-, and metabolomics-related characteristics of population-based cancer metabolomics studies.

Study			Population					Metabolomics			
Author year	Study design	Cancer type	Study setting	Cancer cases/diseased controls/healthy controls	Age range	Reported race	Reported Hispanic ethnicity	Aim	Approach	Analytic platform	Biospecimen
Adams 2019 (79)	Nested case–control study	Prostate	United Kingdom	2,291/0/2,661	18+	NR	NR	Cancer risk estimation using incident cases	Semi-targeted	NMR	Serum
Assi 2015 (80)	Nested case–control study	Liver and intrahepatic bile duct	United Kingdom, Denmark, Germany, Greece, Italy, Netherlands, Spain, Sweden	114/0/222	18+	NR	NR	Biomarker of exposure and cancer risk estimation using incident cases	Untargeted	NMR	Serum
Assi 2018 (81)	Nested case–control study	Liver and intrahepatic bile duct	United Kingdom, Denmark, France, Germany, Greece, Italy, Netherlands, Norway, Spain, Sweden	147/0/147	18+	NR	NR	Biomarker of exposure and cancer risk estimation using incident cases	Semi-targeted	LC-MS	Serum
Assi 2018 (82)	Nested case–control study	Liver and intrahepatic bile duct	United Kingdom, Denmark, Germany, Greece, Italy, Netherlands, Spain, Sweden	147/0/147	18+	NR	NR	Biomarker of exposure and cancer risk estimation using incident cases	Semi-targeted	LC-MS	Serum
Battini 2017 (40)	Case-only study	Pancreas	France	106/0/0	NR	NR	NR	Biomarkers of survival	Semi-targeted	NMR	Tissue
Björkblom 2016 (83)	Nested case–control study	Brain and other nervous system	Norway	110/0/110	18+	NR	NR	Cancer risk estimation using incident cases	Untargeted	GC-MS	Serum
Bro 2015 (84)	Nested case–control study	Breast	Denmark	838/0/838	18+	NR	NR	Cancer risk estimation using incident cases	Untargeted	NMR	Plasma
Bruzzone 2020 (48)	Cross-sectional study	Prostate	Spain	453/202/0	18+	NR	NR	Disease differentiation: cases vs. controls	Untargeted	NMR	Urine
Budczies 2012 (36)	Cross-sectional study	Breast	Germany	184/0/42	18+	NR	NR	Biomarkers of disease diagnosis and survival	Untargeted	GC-MS	Tissue
Cross 2014 (85)	Nested case–control study	Colorectal	United States	255/0/254	18+	Black or African American, White, Other^a^	NR	Biomarker of exposure and cancer risk estimation using incident cases	Semi-targeted	GC-MS, LC-MS	Serum
Cross 2014 (86)	Nested case–control study	Colorectal	United States	254/0/254	18+	Black or African American, White, Other^a^	NR	Cancer risk estimation using incident cases	Semi-targeted	GC-MS, LC-MS	Serum
Deng 2019 (46)	Case-control study	Colorectal	United States, Canada	171/0/171	18+	NR	NR	Cancer risk estimation using incident cases	Semi-targeted	LC-MS	Urine
Dickerman 2020 (87)	Nested case–control study	Prostate	United States	212/0/212	18+	White	NR	Biomarker of exposure and cancer risk estimation using incident cases	Semi-targeted	LC-MS	Plasma
Farshidfar 2016 (88)	Nested case–control study	Colorectal	Canada	222/0/156	18+	NR	NR	Biomarkers of disease diagnosis	Untargeted	GC-MS	Serum
Fest 2019 (89)	Other^b^	Pancreas	Estonia, Finland, Netherlands, Norway	389/0/946	18+	NR	NR	Cancer risk estimation using incident cases	Semi-targeted	NMR	Serum
Gaudet 2012 (90)	Case–control study	Uterus	Poland	250/0/250	18+	NR	NR	Cancer risk estimation using prevalent cases	Semi-targeted	GC-MS	Serum
Geijsen 2019 (91)	Case–control study	Colorectal	Austria, Germany	180/0/153	18+	White	NR	Cancer risk estimation using prevalent cases	Untargeted	LC-MS	Plasma
Guertin 2015 (92)	Nested case–control study	Colorectal	United States	251/0/247	18+	White	NR	Biomarker of exposure and cancer risk estimation using incident cases	Semi-targeted	GC-MS, LC-MS	Serum
Hadi 2017 (93)	Case–control study	Breast	Pakistan	152/0/155	18+	NR	NR	Disease differentiation: cases vs. controls	Untargeted	GC-MS	Serum
Hakimi 2016 (38)	Case-only study	Kidney and renal pelvis	United States	138/0/0	18+	Asian, Black or African American, White, Other^a^	NR	Descriptive study: progression/survival	Semi-targeted	GC-MS, LC-MS	Tissue
Han 2020 (39)	Case-only study	Liver and intrahepatic bile duct	China	156/0/0	18+	NR	NR	Biomarkers of survival	Untargeted	LC-MS	Tissue
Hao 2020 (94)	Case-only study	Lung and bronchus	NR	439/0/0	18+	NR	NR	Risk of recurrence or death among cancer survivors	Untargeted	LC-MS, NMR	Serum
Hasim 2012 (47)	Case–control study	Esophagus	China	108/0/40	18+	NR	NR	Disease differentiation: cases vs. controls	Untargeted	NMR	Plasma, Urine
His 2019 (95)	Nested case–control study	Breast	United Kingdom, Denmark, France, Germany, Greece, Italy, Netherlands, Norway, Spain, Sweden	1,624/0/1,624	18+	NR	NR	Cancer risk estimation using incident cases	Semi-targeted	LC-MS	Plasma
Huang 2016 (29)	Nested case–control study	Prostate	United States	380/0/380	18+	Black or African American, White, Other^a^	Not Hispanic or Latino	Cancer risk estimation using incident cases	Semi-targeted	GC-MS, LC-MS	Serum
Huang 2016 (32)	Case–control study	Breast	United States	106/0/61	18+	American Indian or Alaska Native, Asian, Black or African American, White, Other^a^	Hispanic or Latino	Biomarkers of disease diagnosis	Untargeted	GC-MS, LC-MS	Plasma, Serum
Huang 2019 (96)	Nested case–control study	Prostate	Finland	523/0/523	18+	White	NR	Cancer risk estimation using incident cases	Semi-targeted	LC-MS	Serum
Huang 2020 (97)	Case–control study	Lung and bronchus	China	200/0/200	18+	NR	NR	Biomarkers of disease diagnosis	Untargeted	Other^c^	Serum
Huang 2020 (98)	Nested case–control study	Liver and intrahepatic bile duct	Taiwan	109/0/107	18+	NR	NR	Cancer risk estimation using incident cases	Semi-targeted	NMR	Plasma
Jobard 2021 (99)	Nested case–control study	Breast	France	791/0/791	18+	NR	NR	Cancer risk estimation using incident cases	Untargeted	NMR	Plasma
Kaji 2020 (42)	Case-only study	Stomach	Japan	140/0/0	18+	NR	NR	Risk of recurrence or death among cancer survivors	Untargeted	CE-MS	Tissue
Kliemann 2021 (100)	Nested case–control study	Colorectal, uterus	United Kingdom, Denmark, France, Germany, Italy, Netherlands, Norway, Spain, Sweden	1,058^d^/0/1,071	18+	NR	NR	Cancer risk estimation using incident cases	Semi-targeted	LC-MS	Plasma, Serum
Kühn 2016 (101)	Case-cohort study	Breast, colorectal, prostate	Germany	835^e^/0/774	18+	NR	NR	Cancer risk estimation using incident cases	Semi-targeted	LC-MS, Other^f^	Plasma
Lécuyer 2018 (102)	Nested case–control study	Breast	France	206/0/396	18+	NR	NR	Cancer risk estimation using incident cases	Untargeted	NMR	Plasma
Lécuyer 2019 (103)	Nested case–control study	Breast	France	211/0/211	18+	NR	NR	Cancer risk estimation using incident cases	Untargeted	LC-MS	Plasma
Lécuyer 2020 (104)	Nested case–control study	Breast	France	200/0/200	18+	NR	NR	Biomarker of exposure and cancer risk estimation using incident cases	Untargeted	LC-MS	Plasma
Lécuyer 2021 (105)	Nested case–control study	Prostate	France	171/0/171	18+	NR	NR	Cancer risk estimation using incident cases	Untargeted	NMR	Plasma
Li 2016 (106)	Case–control study	Prostate	China	560/0/500	18+	NR	NR	Biomarkers of disease diagnosis	Untargeted	LC-MS	Plasma
Li 2019 (107)	Cross-sectional study	Colorectal	China	120/120/120	18+	NR	NR	Biomarkers of disease diagnosis	Untargeted	LC-MS	Serum
Li 2021 (108)	Case–control study	Colorectal	China	144/110/74	18+	NR	NR	Biomarkers of disease diagnosis	Untargeted	Other^g^	Plasma
Liang 2016 (109)	Case–control study	Liver and intrahepatic bile duct, other^h^	China	176^i^/0/85	18+	NR	NR	Disease differentiation: cases vs. controls	Untargeted	LC-MS	Serum
Liang 2017 (49)	Case–control study	Prostate	China	236/0/233	18+	NR	NR	Cancer risk estimation using prevalent cases	Untargeted	LC-MS	Urine
Loftfield 2020 (110)	Nested case–control study	Liver and intrahepatic bile duct	Finland	221/242/463	18+	NR	NR	Biomarker of exposure and cancer risk estimation using incident cases	Untargeted	LC-MS	Serum
Louis 2016 (111)	Case–control study	Lung and bronchus	Belgium	233/0/226	18+	NR	NR	Cancer risk estimation using prevalent cases	Semi-targeted	NMR	Plasma
Mamtimin 2011 (112)	Case–control study	Breast, cervix uteri, colorectal, esophagus, liver and intrahepatic bile duct, lung and bronchus, stomach, other^j^	China	170^k^/0/50	18+	NR	NR	Disease differentiation: cases vs. controls	Untargeted	NMR	Plasma
Mathé 2014 (30)	Case–control study	Lung and bronchus	United States	469/0/536	18+	Black or African American, White	NR	Biomarkers of disease diagnosis and survival	Untargeted	LC-MS	Urine
McCullough 2021 (113)	Nested case–control study	Colorectal	United States	517/0/517	18+	Black or African American, White, Other^l^	NR	Cancer risk estimation using incident cases	Semi-targeted	LC-MS	Plasma
Meller 2016 (41)	Case-only study	Prostate	Germany	106/0/0	18+	NR	NR	Association study: prognosis/recurrence	Untargeted	GC-MS, LC-MS	Tissue
Men 2020 (45)	Case–control study	Breast	China	106/0/38	18+	NR	NR	Disease differentiation: cases vs. controls	Untargeted	NMR	Urine
Mondul 2015 (25)	Nested case–control study	Prostate	Finland	200/0/200	18+	White^m^	NR	Cancer risk estimation using incident cases	Semi-targeted	GC-MS, LC-MS	Serum
Moore 2018 (33)	Nested case–control study	Breast	United States	621/0/621	18+	White, Other^n^	Not Hispanic or Latino	Biomarker of exposure and cancer risk estimation using incident cases	Semi-targeted	LC-MS	Serum
Moore 2021 (34)	Nested case–control study	Breast	United States	782/0/782	18+	White, Other^l^	Not Hispanic or Latino	Cancer risk estimation using incident cases	Semi-targeted	LC-MS	Serum
Ose 2021 (114)	Case-only study	Colorectal	Austria, Germany, Netherlands	440/0/0	18+	NR	NR	Risk of recurrence or death among cancer survivors	Semi-targeted	LC-MS	Plasma
Petrick 2019 (23)	Case–control study	Leukemia	United States	332/0/324	0–18	NR	Hispanic or Latino, Not Hispanic or Latino	Disease differentiation: cases vs. controls	Untargeted	LC-MS	Other^o^
Piyarathna 2018 (35)	Case-only study	Bladder	United States, Germany	165/0/0	18+	NR	NR	Cancer progression/natural history	Untargeted	LC-MS	Tissue
Röhnisch 2020 (115)	Nested case–control study	Prostate	Sweden	777/0/777	18+	NR	NR	Cancer risk estimation using incident cases	Semi-targeted	LC-MS, NMR	Plasma
Ros-Mazurczyk 2017 (116)	Case-control study	Lung and bronchus	Poland	100/0/300	18+	NR	NR	Cancer risk estimation using prevalent cases	Untargeted	LC-MS	Serum
Schmidt 2017 (117)	Nested case–control study	Prostate	United Kingdom, Germany, Greece, Italy, Netherlands, Spain	1,077/0/1,077	18+	NR	NR	Cancer risk estimation using incident cases	Semi-targeted	LC-MS	Plasma
Schmidt 2020 (118)	Nested case–control study	Prostate	United Kingdom, Germany, Greece, Italy, Netherlands, Spain	3,057/0/3,057	18+	NR	NR	Cancer risk estimation using incident cases	Semi-targeted	LC-MS	Plasma
Seow 2019 (26)	Nested case–control study	Lung and bronchus	China	275/0/289	18+	Asian^p^	NR	Cancer risk estimation using incident cases	Untargeted	LC-MS, NMR	Urine
Shu 2018 (119)	Nested case–control study	Pancreas	China	226/0/226	18+	NR	NR	Cancer risk estimation using incident cases	Untargeted	GC-MS, LC-MS	Plasma
Shu 2018 (27)	Nested case–control study	Colorectal	China	245/0/245	18+	Asian^p^	NR	Cancer risk estimation using incident cases	Untargeted	GC-MS, LC-MS	Plasma
Stepien 2021 (120)	Nested case–control study	Liver and intrahepatic bile duct	United Kingdom, Denmark, France, Germany, Greece, Italy, Netherlands, Norway, Spain, Sweden	129/0/129	18+	NR	NR	Cancer risk estimation using incident cases	Untargeted	LC-MS	Serum
Stolzenberg-Solomon 2020 (121)	Nested case–control study	Pancreas	United States, Finland	479/0/479	18+	Asian, Black or African American, White, Other^a^	NR	Cancer risk estimation using incident cases	Semi-targeted	GC-MS, LC-MS	Serum
Su 2019 (122)	Nested case–control study	Liver and intrahepatic bile duct	China	134/0/134	18+	NR	NR	Biomarkers of disease diagnosis	Semi-targeted	LC-MS	Serum
Sun 2019 (37)	Case-only study	Esophagus	China	256/0/0	NR	NR	NR	Disease differentiation: tumor vs. non-tumor tissue	Untargeted	Other^q^	Tissue
Vanhove 2018 (123)	Case–control study	Lung and bronchus	Belgium	269/108/347	18+	NR	NR	Biomarkers of disease diagnosis	Semi-targeted	NMR	Plasma
Wang 2016 (43)	Case-only study	Stomach	China	125/0/54	18+	NR	NR	Biomarkers of disease diagnosis	Untargeted	NMR	Tissue
Wang 2021 (124)	Case–cohort study	Prostate	United States	241/0/347	18+	White, Other^r^	NR	Cancer risk estimation using incident cases	Semi-targeted	LC-MS	Plasma
Wei 2021 (125)	Cross-sectional study	Esophagus	China	207/0/257	18+	NR	NR	Biomarker of exposure and cancer risk estimation using prevalent cases	Untargeted	LC-MS	Serum
Wikoff 2015 (126)	Nested case–control study	Lung and bronchus	United States	100/0/199	18+	Black or African American, White, Other^a^	NR	Cancer risk estimation using incident cases	Untargeted	LC-MS	Serum
Wilson 2013 (24)	Case-only study	Brain and other nervous system	United Kingdom	115/0/0	0–18	NR	NR	Risk of recurrence or death among cancer survivors	Semi-targeted	Other^s^	Tissue
Yamakawa 2017 (44)	Cohort study	Stomach	Japan	103/0/0	18+	NR	NR	Risk of recurrence or death among cancer survivors	Untargeted	CE-MS	Tissue
Yi 2014 (28)	Cross-sectional study	Oral cavity and pharynx	China	100/0/100	18+	Asian^t^	NR	Disease differentiation: cases vs. controls	Untargeted	GC-MS	Serum
Zeleznik 2020 (127)	Nested case–control study	Ovary	United States	252/0/252	18+	NR	NR	Cancer risk estimation using incident cases	Semi-targeted	LC-MS	Plasma
Zhang 2020 (128)	Cross-sectional study	Colorectal	China	539/73/0	18+	NR	NR	Biomarkers of disease diagnosis	Untargeted	LC-MS	Plasma
Zhao 2019 (31)	Case–control study	Breast	United States	134/0/57	18+	Black or African American	Hispanic or Latino, Not Hispanic or Latino	Cancer risk estimation using prevalent cases	Semi-targeted	GC-MS, LC-MS	Plasma

Abbreviation: NR, not reported.

^a^Other race groups not specified.

^b^meta-analysis of multiple nested case–control studies.

^c^ferric particle-assisted laser desorption/ionization mass spectrometry.

^d^colorectal cancer = 423, endometrial cancer = 635.

^e^breast cancer = 362, colorectal cancer = 163, prostate cancer = 310.

^f^flow injection analysis-tandem mass spectrometry.

^g^metal-organic framework/platform hybrid-assisted laser desorption/ionization mass spectrometry.

^h^extrahepatic cholangiocarcinoma.

^i^extrahepatic cholangiocarcinoma = 90, intrahepatic cholangiocarcinoma = 86.

^j^leucoma and other not specified.

^k^female breast cancer = 13, cervix cancer = 17, esophageal cancer = 26, hepatoma = 8, leucoma = 10, lung = 26, rectal = 7, stomach = 23, other not specified = 40.

^l^other race groups not specified and unknown.

^m^Finnish.

^n^other race groups not specified and missing.

^o^neonatal blood spots.

^p^Chinese.

^q^airflow-assisted desorption electrospray ionization mass spectrometry imaging.

^r^non-White unspecified.

^s^1H magnetic resonance spectroscopy.

^t^Han Chinese.

In general, the number of cancer epidemiology studies employing metabolomics has increased over time from 2011 through June 2021 (Supplementary Fig. S1).

**Figure 2.**
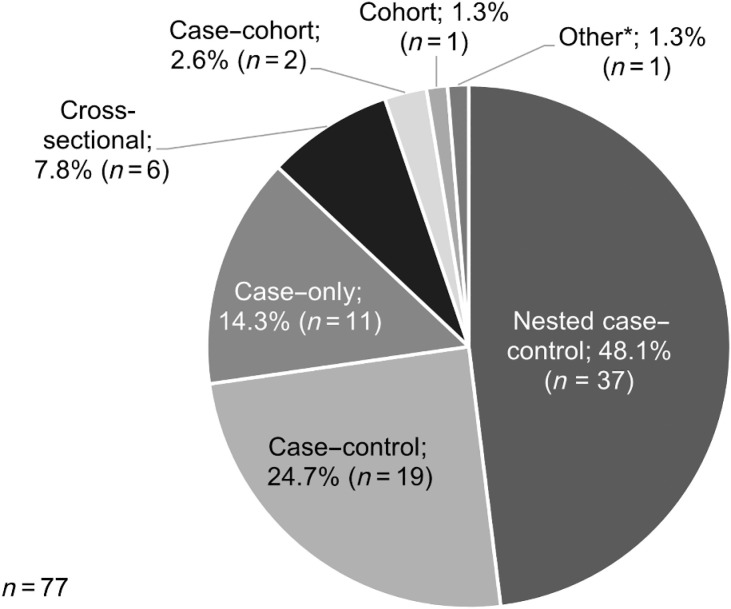
Pie chart displays the breakdown of study design types used in metabolomic epidemiology studies of cancer. *Other includes meta-analysis of multiple nested case–control studies.

Figure 2 shows the breakdown of study design types used in metabolomic epidemiology studies of cancer. The most common study design used was a nested case–control study design (*n* = 37, 48.1%). Case–control studies (*n* = 19, 24.7%) and case-only studies (*n* = 11, 14.3%) were the next two most used.

**Figure 3.**
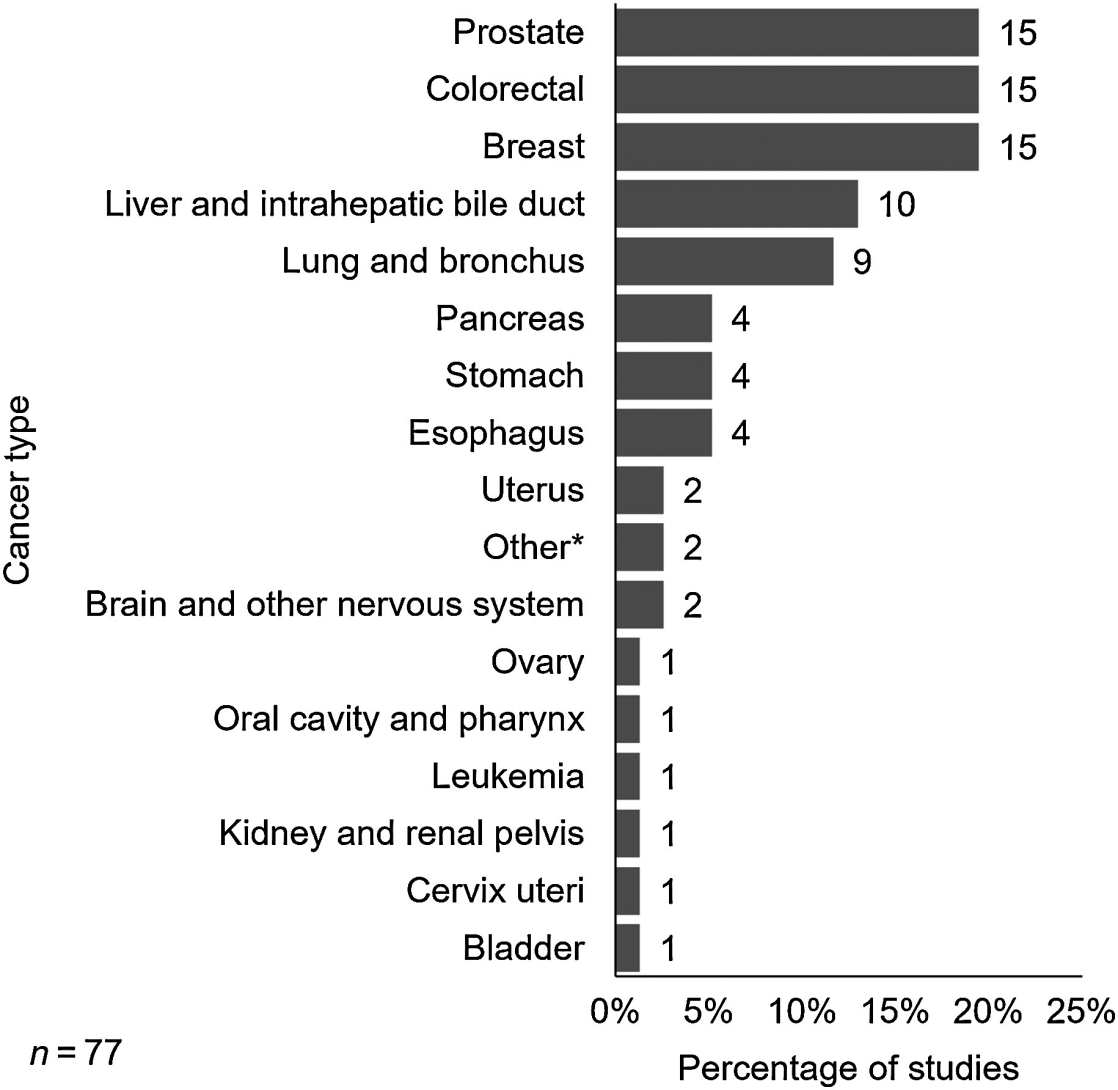
Bar graph depicts cancer types studied in the metabolomic epidemiology literature. *Other includes extrahepatic cholangiocarcinoma, leucoma, and other not specified.

The primary cancer types investigated were colorectal (*n* = 15, 19.5%), prostate (*n* = 15, 19.5%), and female breast (*n* = 15, 19.5%; Fig. 3). The studies included in this review examined 19 different cancer types.

Most studies investigated adult-onset cancer (*n* = 73, 94.8%; Table 1). Two studies (2.6%) included subjects under 18 years of age to study pediatric cancers, namely, acute lymphoblastic leukemia (23) and pediatric brain tumors (24). Two studies (2.6%) did not report participant age information; however, these studies focused on adult-onset cancers (pancreas and esophagus).

The geographic distribution of studies is displayed in Supplementary Fig. S2. Most studies were conducted in European countries, including Germany (*n* = 14, 18.2%), France (*n* = 10, 13.0%), the Netherlands (*n* = 10, 13.0%), and the United Kingdom (*n* = 10, 13.0%). Studies were also conducted in the United States (*n* = 19, 24.7%) and China (*n* = 19, 24.7%). One study (1.3%) was missing study setting data.

Only 27.3% (*n* = 21) of studies reported on race (Table 1; Supplementary Fig. S3). In studies reporting race, 17 reported White participants, 11 reported other race groups (i.e., other race groups not specified, unknown, missing, and non-White unspecified), 10 reported Black or African American participants, 6 reported Asian participants, and 1 reported American Indian or Alaska Native participants (Supplementary Fig. S3). Of the 17 studies reporting White participants, only the study by Mondul and colleagues (25) specified that participants were Finnish. Of the six studies reporting Asian participants, three studies specified that participants were Chinese (26–28). Yi and colleagues (28) provided the distinction that all participants were Han Chinese. Three studies compared race or ethnicity groups (29–31). Mathé and colleagues (30) investigated urine metabolite predictors of lung cancer among Black and White participants. Stratified analyses by self-reported race revealed cortisol sulfate to have the strongest association with survival in Black participants. Huang and colleagues (29) performed serum metabolomics in a nested case–control design to examine prostate cancer risk in a sample population that included non-Hispanic White men, Black men, and men of other races. Their analysis by race did not reveal substantial differences in serum metabolites of overall prostate cancer risk between race groups, although sample sizes for non-White men were small. Zhao and colleagues (31) looked at plasma metabolites and breast cancer risk in Hispanic and non-Hispanic Black women. The authors did not find differences in metabolite profiles between Black and Hispanic women among the controls but acknowledged that they did not have adequate power to detect a difference. Five additional studies reported Hispanic ethnicity of their participants but did not make comparisons between ethnic groups (23, 29, 32–34).

All studies included in this review had a minimum of 100 cancer cases in each analysis stratification of the main analysis. Most studies (*n* = 54, 70.1%) included fewer than 300 cancer cases and 6.5% (*n* = 5) included over 1,000 cancer cases (Supplementary Fig. S4).

Cancer types that included on average greater than 300 cases across all studies were prostate (*n* = 706; 15 studies), breast (*n* = 451; 14 studies), uterus (*n* = 443; 2 studies), and leukemia (*n* = 332; 1 study; Supplementary Fig. S5).

**Figure 4.**
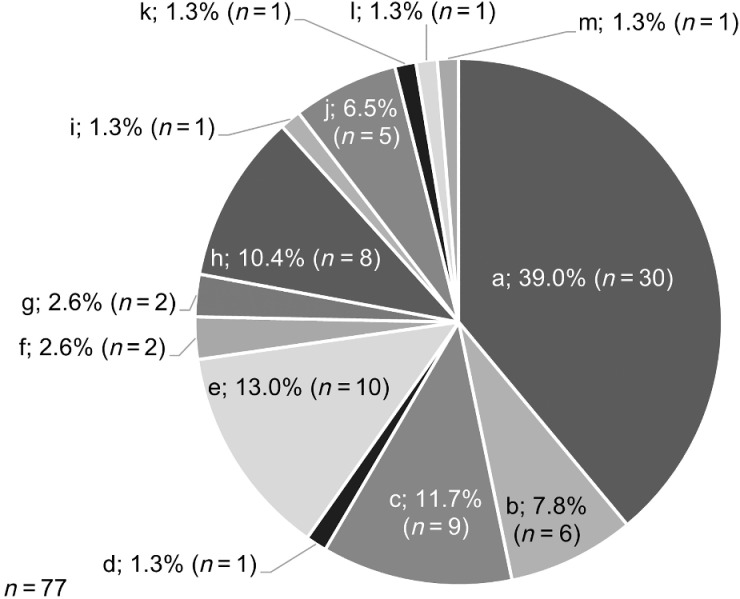
Pie chart shows the breakdown of metabolomics-specific aims of included metabolomic epidemiology studies of cancer. (a) Cancer risk estimation using incident cases; (b) cancer risk estimation using prevalent cases; (c) biomarker of exposure and cancer risk estimation using incident cases; (d) biomarker of exposure and cancer risk estimation using prevalent cases; (e) biomarkers of disease diagnosis; (f) biomarkers of survival; (g) biomarkers of disease diagnosis and survival; (h) disease differentiation: cases vs. controls; (i) disease differentiation: tumor vs. non-tumor tissue; (j) risk of recurrence or death among cancer survivors; (k) cancer progression/natural history; (l) association study: prognosis/recurrence; (m) descriptive study: progression/survival.

Figure 4 displays the metabolomics-specific aims pursued by eligible studies. The majority of studies used metabolomics to estimate cancer risk (*n* = 46, 59.8%). Of these studies, 30 estimated cancer risk using incident cases, 6 estimated cancer risk using prevalent cases, 9 examined biomarkers of exposure and cancer risk using incident cases, and 1 study examined biomarkers of exposure and cancer risk using prevalent cases. Metabolomics was used in other studies to primarily identify biomarkers of disease diagnosis (*n* = 10, 13.0%) and perform disease differentiation between cases and controls (*n* = 8, 10.4%).

Serum (*n* = 31, 40.3%) and plasma (*n* = 30, 39.0%) were the most used biospecimens (Table 1). Eleven studies (14.3%) performed metabolomics on tissue to study cancers of the bladder (35), brain and other nervous system (24), breast (36), esophagus (37), kidney and renal pelvis (38), liver and intrahepatic bile duct (39), pancreas (40), prostate (41), and stomach (refs. 42–44; Table 1). Seven studies (9.1%) used urine samples to study breast (45), colorectal (46), esophageal (47), lung and bronchus (26, 30), and prostate cancers (refs. 48, 49; Table 1). Petrick and colleagues (23) used neonatal blood spots to study acute lymphoblastic leukemia.

The proportions of untargeted and semi-targeted studies were similar, 55.8% (*n* = 43) and 44.2% (*n* = 34), respectively (Table 1). Supplementary Table S1 displays the metabolite super-pathways assessed in the 34 semi-targeted studies. All studies examined metabolites belonging to the lipid super-pathway. Following lipids, the second most covered super-pathway of metabolites was amino acids.

The most frequently used analytic platforms were liquid chromatography–tandem mass spectrometry (LC-MS; *n* = 49, 63.6%), NMR spectroscopy (*n* = 19, 24.7%), and gas chromatography–tandem mass spectrometry (GC-MS; *n* = 18, 23.4%; Table 1).

### Synthesis of results

The number of cancer epidemiology studies using metabolomics assays has increased over time from 2011 through June 2021. Colorectal, prostate, and female breast cancers were the most well-studied cancers. Studies were geographically diverse, but few studies reported on race or ethnicity, and those that did reported a majority of White participants. Most studies included fewer than 300 cancer cases in their main analysis. Most studies enrolled adult participants, used a nested case–control design to estimate metabolite-cancer risk, and used an LC-MS untargeted or semi-targeted approach to measure metabolites in either serum or plasma. Most studies measured lipids and amino acids.

## Discussion

### Summary of evidence

We identified 77 population-based cancer metabolomics research studies, published between January 1998 and June 2021, that investigated 19 different cancer types. Although studies were conducted across diverse geographic settings, our findings indicate a need for standardized reporting of race and ethnicity of study participants, as well as more racially/ethnically diverse study populations. Our findings also indicate trends in metabolomic epidemiology for prospective designs to assess cancer risk, blood-based biospecimens, mass spectrometry–based platforms, and untargeted metabolomics. Studies that used a semi-targeted strategy largely covered lipid and amino acid super-pathways. Furthermore, sample sizes of included metabolomic epidemiology studies of cancer emphasize the need for larger studies in the field.

Most epidemiologic studies have used metabolomics to gain insight into cancer etiology. Accordingly, they used a prospective case–control design nested in large cohorts that collected blood or other biospecimens at study baseline.

Colorectal, prostate, and female breast cancers were the most studied cancers and are also among the most common cancers worldwide (50), which enables researchers to more easily recruit or ascertain cases in epidemiologic studies. Etiologic studies of rare cancers are more challenging as low incidence rates for rare cancers pose as limitations to case recruitment and require very large sample sizes and extended follow-up to ascertain sufficient case numbers for analysis in prospective studies. Although rare cancers individually have low incidence rates, they collectively account for 20% to 25% of all oncology patients (51). Patients with rare cancers often have relatively worse outcomes for reasons including delayed diagnoses, less effective standard treatments, reduced access to clinical expertise, and gaps in funding for preclinical and clinical research (51). Metabolomics studies investigating rare cancers may improve their diagnosis, prognosis, and treatment.

Most studies performed metabolomics on serum or plasma as many mature cohort studies collected baseline blood. However, a broader range of biospecimen types (e.g., stool, tumor tissue) collected in ongoing and future cohorts may further increase discovery potential in metabolomic epidemiology studies.

Mass spectrometry–based analytic platforms, specifically LC-MS, were most often used by metabolomic epidemiology studies investigating cancer. Mass spectrometry–based metabolomics has historically been used in cancer research for early detection and screening applications (2). Within the subset of studies that used LC-MS in a semi-targeted approach (*n* = 26), the majority conducted their metabolomic profiling through Metabolon, Inc. and Biocrates Life Sciences (*n* = 22; Supplementary Fig. S6). Collaborations with fee-for-service industry partners provides an opportunity for epidemiologists and researchers without their own metabolomics laboratories to pursue metabolomics experiments (52). These platforms are most likely capturing the metabolites that align with the hypotheses of these studies. Studies that performed semi-targeted metabolomics primarily covered lipid and amino acid metabolism. The wide coverage of lipids is likely due to lipids being chemically well-studied. Furthermore, alterations in lipid and amino acid metabolism have been identified as hallmarks of cancer (2). Lipids assume functions in a variety of cancer processes, such as angiogenesis, cell growth, proliferation, survival, migration, invasion, and metastasis (53–55). Among the ways amino acid metabolism supports cancer cell growth and proliferation include providing materials for macromolecule (e.g., protein, lipid, and nucleic acid) synthesis and supplying alternative energy sources through anaplerotic reactions that feed the citric acid cycle in times of glucose deficiency (2, 56, 57). Considering that lipid and amino acid metabolism are integral to cancer metabolism, studying the metabolites that fall under these pathways may provide valuable insights that lead to improvements in cancer diagnosis, prognosis, and therapy development.

Two key gaps in the population-based cancer metabolomics literature were identified in this scoping review: (i) the need for standardized race and ethnicity reporting and (ii) the need for larger studies.

In this review, we found that a minority of studies reported on study participant race and ethnicity. Across the studies that did report these data, few reported subgroup information, and there was variation in where in the manuscript (e.g., methods, results, supplementary data) race and ethnicity information was reported. These findings demonstrate a need in the field to outline reporting standards for participant race and ethnicity information. Lin and Kelsey (58) explain that race and ethnicity data have potential utility in epidemiologic research to elucidate disease etiology, reveal the roles and interactions of genes and environment in disease, identify subgroups experiencing unequal care, assess population-specific conceptualizations of disease factors for developing tailored interventions, and study within-group biological variations. Clear reporting of participant race, ethnicity, and other sociodemographic information can aid researchers in determining study generalizability and uncovering health disparities and inequities (59), which is important when considering cancers with known racial and ethnic disparities, such as higher incidence of triple-negative breast cancer in Black women and liver cancer in Asian individuals. Although we did observe more consistent reporting of study setting, explicit reporting of race and ethnicity is needed to prevent the inaccurate conflation of geographic setting data as proxies for race and ethnicity by readers. The international medical and scientific publishing community has outlined recommendations for race and ethnicity reporting in the scientific literature (59–62). Perhaps initiatives can be spurred from within the metabolomic epidemiology community to draft and adopt guidance specific to the field.

Additionally, diverse representation is needed in metabolomic epidemiology studies. Lack of diversity has been well documented in genomics and the Eurocentric focus in the field has negative consequences including lack of generalizability, as well as poor replicability and accuracy of results in other populations (63, 64). Metabolomics researchers can learn from genomics and prioritize diverse study representation in order to reflect diversity more accurately within and across populations. In mature cancer cohorts, case numbers for rare cancers and some minority populations can be limited. As we saw in the present review, most studies examined sample sizes of fewer than 300 cancer cases.

Sample size affects statistical power or the ability to detect a statistically significant association (65). Depending on the level of variability in metabolite levels that arise due to between-subject, within-subject, and technical variability a larger sample size, with a sufficient number of cases, may be required (66, 67). High between-subject variability is good (i.e., large effect size), whereas high within-subject and technical variability decrease study power (66, 67). Sampson and colleagues (67) reported that to detect associations between metabolites and disease in a case–control design, population-based metabolomics studies require large sample sizes of 1,000 or more subjects, assuming 1:1 matching and a single measurement; however, they also demonstrated that incorporating serial measurements improved statistical power by reducing within-subject variability. Although some epidemiologic studies, particularly cohort studies, have collected and stored serial blood samples, few metabolomics analyses nested in these larger studies have incorporated them. In fact, only two studies reviewed included serial samples (Supplementary Table S2). Power calculations performed by Nicholson and colleagues (68) estimate that sample sizes of a few thousand are sufficient for identifying disease-predictive metabolite levels. To detect the likely moderate to weak effect sizes between an individual metabolite and cancer risk, large sample sizes are needed (67). Further exacerbating the sample size requirements is the issue of multiple comparisons because epidemiologic studies using an untargeted or semi-targeted metabolomics approach analyze hundreds of metabolites or thousands of metabolite features in relation to a disease outcome; thus, multiple testing correction is needed to limit false positives (11). While focusing on metabolites within a single biological pathway could preserve study power, the tradeoff would be the omittance of valuable data (11).

The upstream solution of establishing larger, more diverse cohorts is important. The NIH's All of Us Research Program and National Cancer Institute's Connect for Cancer Prevention Study are examples of newly established prospective cohorts that are focused on recruiting large sample sizes (1,000,000+ and 200,000 participants, respectively) and participants from diverse backgrounds and geographic locations that have been historically underrepresented in cancer research (69–71). Although this effort is necessary, such large cohorts cost tens of millions of dollars to set up and take decades to mature (72, 73). A potentially more tractable recommendation, then, is to design pooling and consortia efforts. Collaborative consortia offer the opportunity for researchers to achieve larger sample sizes and increased statistical power through pooling of metabolomics data across studies (74). The COnsortium of METabolomics Studies (COMETS) is an example of such a consortium where researchers are currently developing methods to harmonize existing data and assaying new samples across multiple cohorts (6, 8, 75, 76). COMETS is comprised of prospective cohorts with blood metabolomics data, acquired using NMR or MS, on 100 or more participants who are followed longitudinally for disease outcomes (6, 8). With over 70 cohorts from Asia, Europe, North America, and South America to date, COMETS can support large population-based studies with increasing geographic and demographic diversity (6, 8).

The first successful pooling project based on COMETS performed pooled analyses on data from greater than 32,000 participants (from 16 and 17 population-based studies) from the United States, Europe, and Asia to examine associations of metabolites (i.e., circulating trimethylamine N-oxide and choline and its related metabolites) with cardiometabolic biomarkers and with dietary and nondietary factors (77, 78). For pooling studies that are harmonizing existing data across multiple cohorts, funding needs only to cover the analytic work which minimizes cost. COMETS Analytics was developed to support consortia-based analyses through a standardized, federated approach for meta-analysis of metabolomics data (75, 76).

For pooling projects that assay new samples, funding needs to consider common samples for quality control (QC). COMETS has developed a reference set of 30 samples (serum, EDTA plasma, or heparinized plasma) and three pooled QC samples to facilitate metabolite level comparisons and promote pooling analyses (6, 8). Incorporating serial samples, when available, also has the potential to reduce metabolite measure variability and improve statistical power.

An additional downstream solution to address issues of sample size and statistical power would be to follow up promising leads from untargeted and semi-targeted metabolomics studies with targeted analyses for further validation of findings. Targeted studies can also be solely performed in cohort studies if the hypothesis warrants it. Targeted studies are hypothesis driven, do not contend with issues of multiple comparisons, and the measurements are quantified, therefore making them less vulnerable to instrument variation.

### Limitations

This review has several limitations. Due to a lack of language translation capability, we only included studies published in English. We also used race and ethnicity categories outlined by a United States federal organization for our data extraction. Consequently, our results may not fully capture race and ethnic groups, or terminology used in other countries. Additionally, our results cover the published literature up to June 2021 but are reflective of trends in metabolomics and cancer epidemiology over more than two decades. Although grading study design and the appropriate use of statistical methods was beyond the scope of this review, we noted a general need for more standardized methods and reporting of results, which would not only increase transparency but also the ability to compare and reproduce findings.

### Conclusions

This paper presents a comprehensive scoping review of the literature at the intersection of metabolomics, cancer, and epidemiology. The application of metabolomics in an epidemiologic context to study cancer is increasingly feasible and prevalent, with utility in risk estimation, biomarker identification, disease differentiation, and diagnosis and prognosis efforts. As the field continues to advance, researchers should focus on making strides in diverse participant sampling, clear reporting of race and ethnicity, and conducting well-powered studies incorporating more individuals or longitudinal metabolomics data.

## Supplementary Material

Supplementary Figure S1 Supplementary Figure S1 shows metabolomic epidemiology studies of cancer published from January 1998 to June 2021.

Supplementary Figure S2 Supplementary Figure S2 shows geographic distribution of participant recruitment for metabolomic epidemiology studies of cancer.

Supplementary Figure S3 Supplementary Figure S3 shows bar graph displaying the distribution of metabolomic epidemiology studies of cancer reporting race information by race category.

Supplementary Figure S4 Supplementary Figure S4 shows bar graph displaying distribution of metabolomic epidemiology studies of cancer by number of cancer cases recruited.

Supplementary Figure S5 Supplementary Figure S5 shows scatterplot illustrating 80 cancer-metabolomics primary analyses from 77 studies. Studies that had multiple cancer outcomes were considered separately in the analysis.

Supplementary Figure S6 Supplementary Figure S6 shows pie chart displaying breakdown of studies that used an LC-MS semi-targeted approach.

Supplementary Table S1 Supplementary Table S1 shows metabolite super-pathways examined in semi-targeted population-based cancer metabolomics studies.

Supplementary Table S2 Supplementary Table S2 shows additional study design and analysis characteristics of population-based cancer metabolomics studies.
