# Supplementary material for: Characteristics of Cancer Epidemiology Studies That Employ Metabolomics: A Scoping Review
Source: Cancer Epidemiol Biomarkers Prev. 2023 Jul 6;32(9):1130–45. doi: 10.1158/1055-9965.EPI-23-0045 (PMC10472112; doi:10.1158/1055-9965.EPI-23-0045)
Supplement: Supplementary Figure S1 — shows metabolomic epidemiology studies of cancer published from January 1998 to June 2021. [file epi-23-0045_supplementary_figure_s1_suppsf1.pdf]

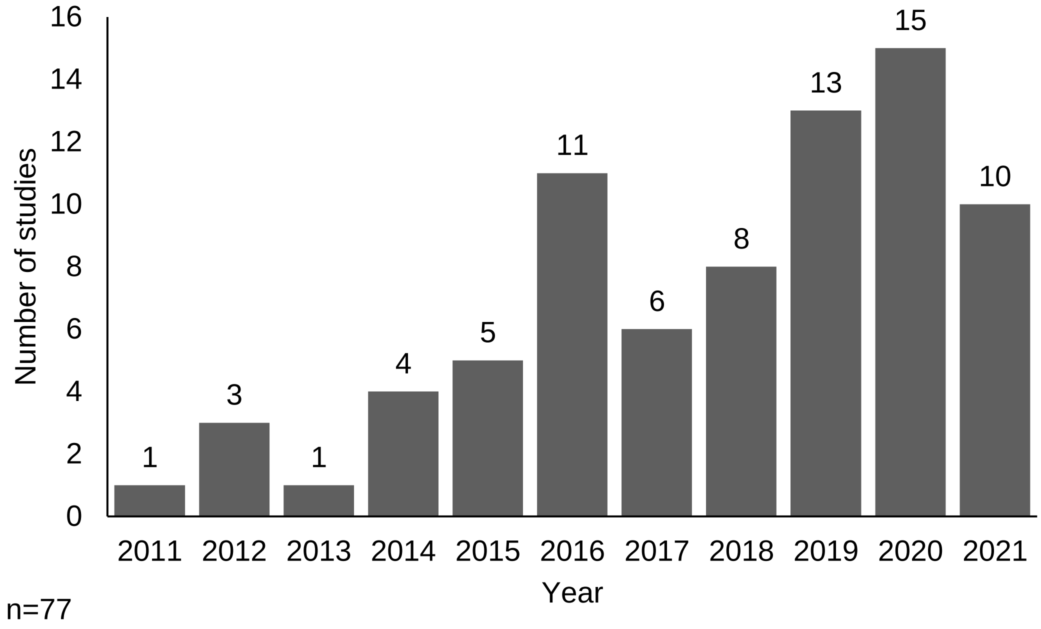

Supplementary Figure S1: Metabolomic epidemiology studies of cancer published from January 1998 to June 2021.
