# Supplementary material for: Characteristics of Cancer Epidemiology Studies That Employ Metabolomics: A Scoping Review
Source: Cancer Epidemiol Biomarkers Prev. 2023 Jul 6;32(9):1130–45. doi: 10.1158/1055-9965.EPI-23-0045 (PMC10472112; doi:10.1158/1055-9965.EPI-23-0045)
Supplement: Supplementary Figure S2 — shows geographic distribution of participant recruitment for metabolomic epidemiology studies of cancer. [file epi-23-0045_supplementary_figure_s2_suppsf2.pdf]

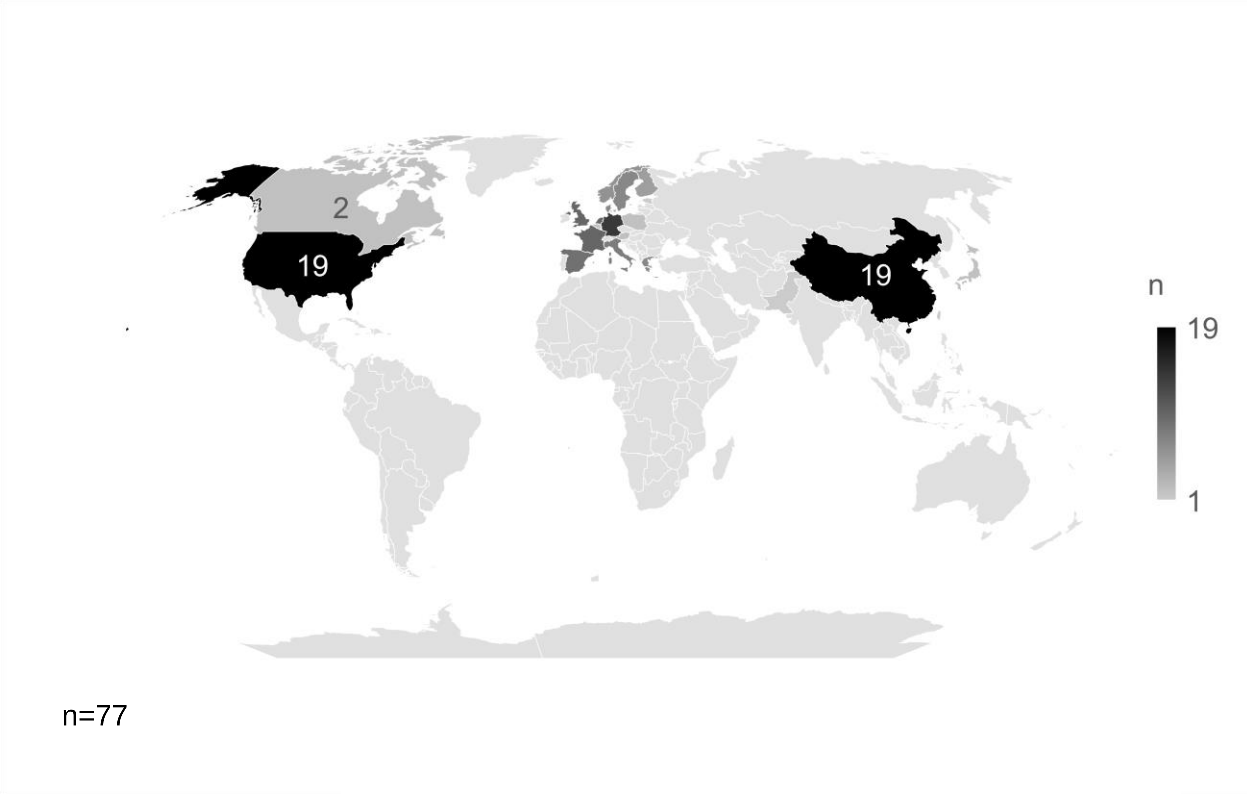

Supplementary Figure S2: Geographic distribution of participant recruitment for metabolomic epidemiology studies of cancer. One study had missing data for study setting.
