# Supplementary material for: Characteristics of Cancer Epidemiology Studies That Employ Metabolomics: A Scoping Review
Source: Cancer Epidemiol Biomarkers Prev. 2023 Jul 6;32(9):1130–45. doi: 10.1158/1055-9965.EPI-23-0045 (PMC10472112; doi:10.1158/1055-9965.EPI-23-0045)
Supplement: Supplementary Figure S3 — shows bar graph displaying the distribution of metabolomic epidemiology studies of cancer reporting race information by race category. [file epi-23-0045_supplementary_figure_s3_suppsf3.pdf]

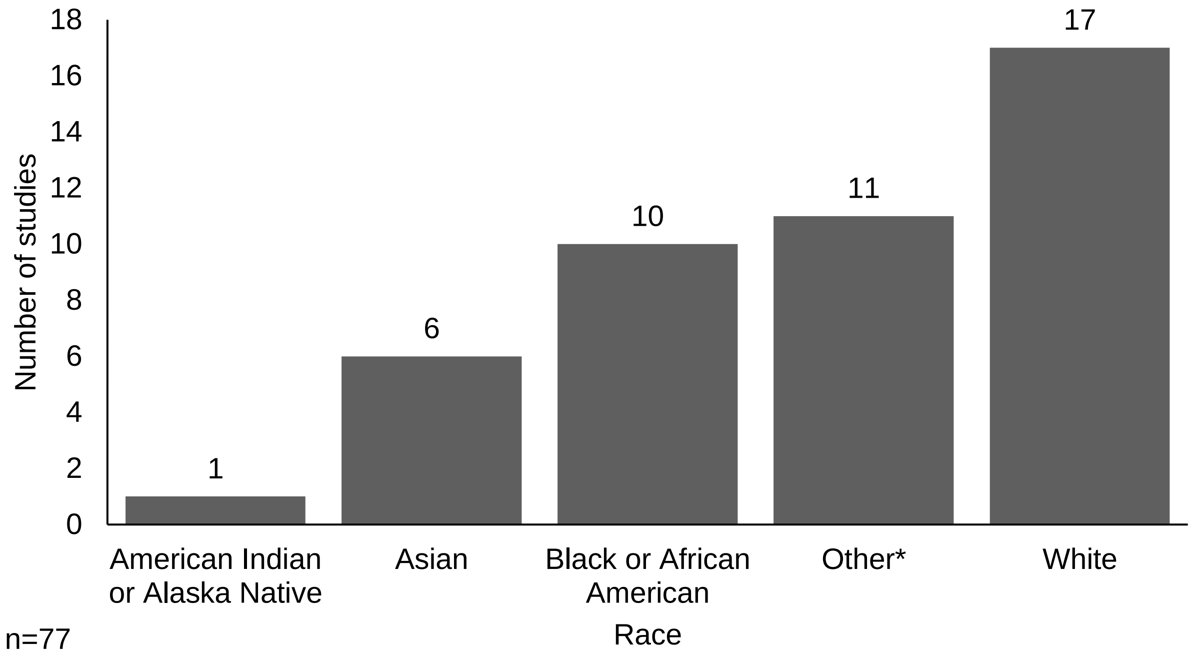

Supplementary Figure S3: Bar graph displays the distribution of metabolomic epidemiology studies of cancer reporting race information by race category. *Other includes other race groups not specified, unknown, missing, and non-White unspecified.
