# Supplementary material for: Characteristics of Cancer Epidemiology Studies That Employ Metabolomics: A Scoping Review
Source: Cancer Epidemiol Biomarkers Prev. 2023 Jul 6;32(9):1130–45. doi: 10.1158/1055-9965.EPI-23-0045 (PMC10472112; doi:10.1158/1055-9965.EPI-23-0045)
Supplement: Supplementary Figure S4 — shows bar graph displaying distribution of metabolomic epidemiology studies of cancer by number of cancer cases recruited. [file epi-23-0045_supplementary_figure_s4_suppsf4.pdf]

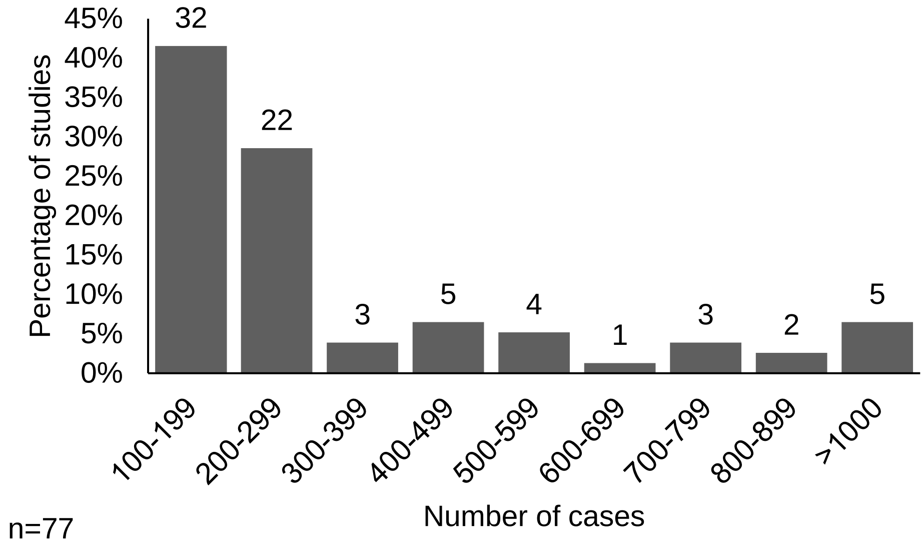

Supplementary Figure S4: Bar graph displays distribution of metabolomic epidemiology studies of cancer by number of cancer cases recruited.
