# Supplementary material for: Characteristics of Cancer Epidemiology Studies That Employ Metabolomics: A Scoping Review
Source: Cancer Epidemiol Biomarkers Prev. 2023 Jul 6;32(9):1130–45. doi: 10.1158/1055-9965.EPI-23-0045 (PMC10472112; doi:10.1158/1055-9965.EPI-23-0045)
Supplement: Supplementary Figure S5 — shows scatterplot illustrating 80 cancer-metabolomics primary analyses from 77 studies. Studies that had multiple cancer outcomes were considered separately in the analysis. [file epi-23-0045_supplementary_figure_s5_suppsf5.pdf]

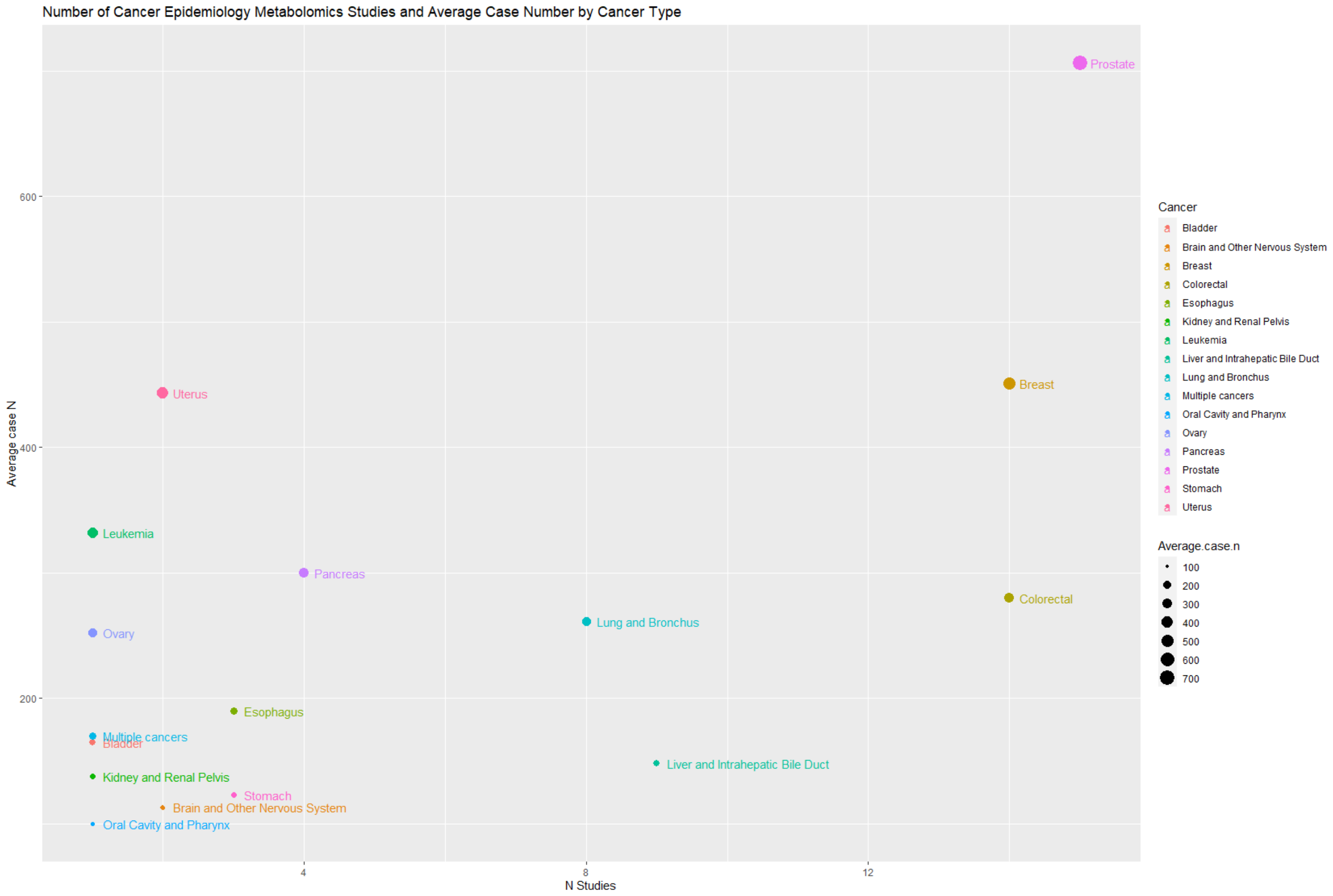

Supplementary Figure S5: Scatterplot illustrates 80 cancer-metabolomics primary analyses from 77 studies. Studies that had multiple cancer outcomes were considered separately in the analysis. Kuhn, et al. was considered as 3 analyses and Kliemann et al. as 2 analyses. X axis = number of studies; Y axis = average number of cases.
