# Supplementary material for: Characteristics of Cancer Epidemiology Studies That Employ Metabolomics: A Scoping Review
Source: Cancer Epidemiol Biomarkers Prev. 2023 Jul 6;32(9):1130–45. doi: 10.1158/1055-9965.EPI-23-0045 (PMC10472112; doi:10.1158/1055-9965.EPI-23-0045)
Supplement: Supplementary Figure S6 — shows pie chart displaying breakdown of studies that used an LC-MS semi-targeted approach. [file epi-23-0045_supplementary_figure_s6_suppsf6.pdf]

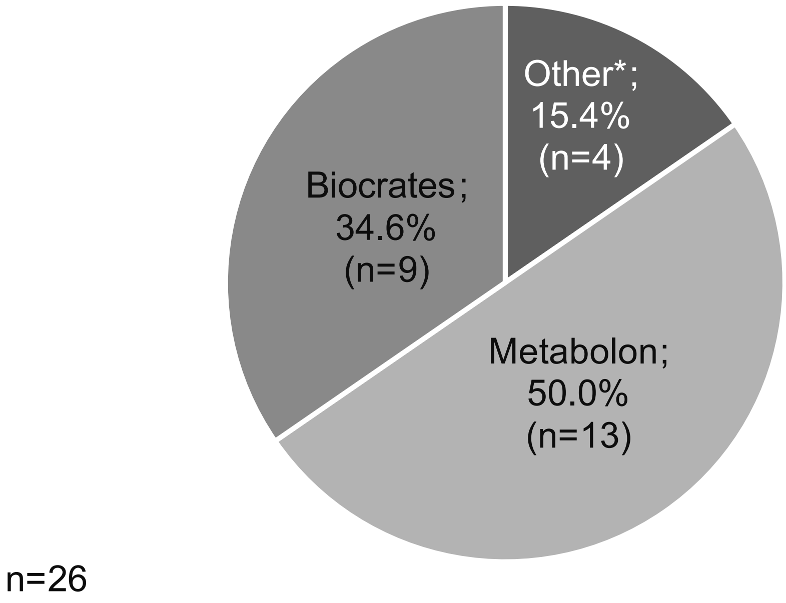

Supplementary Figure S6: Pie chart displays breakdown of studies that used an LC-MS semi-targeted approach. *Other includes Broad Institute, The Metabolomics Innovation Centre, and an unspecified pseudotargeted metabolomics approach.
