# Supplementary material for: Characteristics of Cancer Epidemiology Studies That Employ Metabolomics: A Scoping Review
Source: Cancer Epidemiol Biomarkers Prev. 2023 Jul 6;32(9):1130–45. doi: 10.1158/1055-9965.EPI-23-0045 (PMC10472112; doi:10.1158/1055-9965.EPI-23-0045)
Supplement: Supplementary Table S1 — shows metabolite super-pathways examined in semi-targeted population-based cancer metabolomics studies. [file epi-23-0045_supplementary_table_s1_suppst1.pdf]

Supplementary Table S1: Metabolite super-pathways examined in semi-targeted population-based cancer metabolomics studies.

Author year	Metabolite super-pathways covered
Adams 2019 (79)	amino acids, carbohydrates, energy, lipids
Assi 2018 (81)	amino acids, carbohydrates, lipids, peptides, other
Assi 2018 (82)	amino acids, carbohydrates, lipids, peptides, other
Battini 2017 (40)	amino acids, carbohydrates, energy, lipids, other
Cross 2014 (85)	amino acids, carbohydrates, cofactors and vitamins, energy, lipids, nucleotides, peptides, xenobiotics
Cross 2014 (86)	amino acids, carbohydrates, cofactors and vitamins, energy, lipids, nucleotides, peptides, xenobiotics
Deng 2019 (46)	amino acids, carbohydrates, cofactors and vitamins, energy, lipids, nucleotides, peptides, xenobiotics, other
Dickerman 2020 (87)	amino acids, cofactors and vitamins, lipids, nucleotides
Fest 2019 (89)	amino acids, carbohydrates, energy, lipids
Gaudet 2012 (90)	amino acids, lipids
Guertin 2015 (92)	amino acids, carbohydrates, cofactors and vitamins, energy, lipids, nucleotides, peptides, xenobiotics
Hakimi 2016 (38)	amino acids, carbohydrates, cofactors and vitamins, energy, lipids, nucleotides, peptides, xenobiotics
His 2019 (95)	amino acids, carbohydrates, lipids, peptides, other
Huang 2016 (29)	amino acids, carbohydrates, cofactors and vitamins, energy, lipids, nucleotides, peptides, xenobiotics
Huang 2019 (96)	amino acids, carbohydrates, cofactors and vitamins, energy, lipids, nucleotides, peptides, xenobiotics
Huang 2020 (98)	amino acids, carbohydrates, energy, lipids, other
Kliemann 2021 (100)	amino acids, carbohydrates, lipids, peptides, other
Kühn 2016 (101)	amino acids, carbohydrates, lipids
Louis 2016 (111)	amino acids, carbohydrates, energy, lipids
McCullough 2021 (113)	amino acids, carbohydrates, cofactors and vitamins, energy, lipids, nucleotides, peptides, xenobiotics
Mondul 2015 (25)	amino acids, carbohydrates, cofactors and vitamins, energy, lipids, nucleotides, peptides, xenobiotics
Moore 2018 (33)	amino acids, carbohydrates, cofactors and vitamins, energy, lipids, nucleotides, peptides, xenobiotics
Moore 2021 (34)	amino acids, carbohydrates, cofactors and vitamins, energy, lipids, nucleotides, peptides, xenobiotics
Ose 2021 (114)	amino acids, carbohydrates, lipids, peptides, other
Röhnisch 2020 (115)	amino acids, carbohydrates, lipids, peptides, other
Schmidt 2017 (117)	amino acids, carbohydrates, lipids, peptides, other
Schmidt 2020 (118)	amino acids, carbohydrates, lipids, peptides, other
Stolzenberg-Solomon 2020 (121)	amino acids, carbohydrates, cofactors and vitamins, energy, lipids, nucleotides, peptides, xenobiotics

Su 2019 (122)	amino acids, carbohydrates, cofactors and vitamins, energy, lipids, nucleotides, xenobiotics, other
Vanhove 2018 (123)	amino acids, carbohydrates, energy, lipids
Wang 2021 (124)	amino acids, carbohydrates, cofactors and vitamins, energy, lipids, nucleotides, peptides, xenobiotics
Wilson 2013 (24)	amino acids, carbohydrates, lipids, other
Zeleznik 2020 (127)	lipids
Zhao 2019 (31)	amino acids, carbohydrates, cofactors and vitamins, energy, lipids, nucleotides, peptides, xenobiotics
