# Supplementary material for: Characteristics of Cancer Epidemiology Studies That Employ Metabolomics: A Scoping Review
Source: Cancer Epidemiol Biomarkers Prev. 2023 Jul 6;32(9):1130–45. doi: 10.1158/1055-9965.EPI-23-0045 (PMC10472112; doi:10.1158/1055-9965.EPI-23-0045)
Supplement: Supplementary Table S2 — shows additional study design and analysis characteristics of population-based cancer metabolomics studies. [file epi-23-0045_supplementary_table_s2_suppst2.pdf]

Supplementary Table S2: Additional study design and analysis characteristics of population-based cancer metabolomics studies.[a]

Author year	Serial Samples	Discrimination Statistics[b]	Validation Set[c]
Adams 2019 (23)	N	N	N
Assi 2015 (24)	N	N	N
Assi 2018 (25)	N	N	N
Assi 2018 (26)	N	Y	N
Battini 2017 (27)	N	Y	N
Björkblom 2016 (28)	N	Y	N
Bro 2015 (29)	N	Y	N
Bruzzone 2020 (30)	N	Y	Y
Budczies 2012 (31)	N	N	Y
Cross 2014 (32)	N	N	N
Cross 2014 (33)	N	N	N
Deng 2019 (34)	N	Y	Y
Dickerman 2020 (35)	N	Y	N
Farshidfar 2016 (36)	N	Y	Y
Fest 2019 (37)	N	N	Y
Gaudet 2012 (38)	N	N	N
Geijsen 2019 (39)	N	N	Y
Guertin 2015 (40)	N	N	N
Hadi 2017 (41)	N	Y	Y
Hakimi 2016 (42)	N	N	N
Han 2020 (43)	N	Y	Y
Hao 2020 (44)	N	N	Y
Hasim 2012 (45)	N	Y	N
His 2019 (46)	N	N	N
Huang 2016 (47)	N	N	N
Huang 2016 (48)	N	N	Y
Huang 2019 (49)	N	N	N
Huang 2020 (50)	N	N	Y
Huang 2020 (51)	Y	N	Y
Jobard 2021 (52)	N	Y	N
Kaji 2020 (53)	N	N	N
Kliemann 2021 (54)	N	N	Y
Kühn 2016 (55)	N	N	N
Lécuyer 2018 (56)	N	N	N
Lécuyer 2019 (57)	N	N	N
Lécuyer 2020 (58)	N	N	N
Lécuyer 2021 (59)	N	N	N
Li 2016 (60)	N	Y	N

Li 2019 (61)	N	Y	Y
Li 2021 (62)	N	N	Y
Liang 2016 (63)	N	Y	Y
Liang 2017 (64)	N	Y	Y
Loftfield 2020 (65)	N	N	N
Louis 2016 (66)	N	Y	Y
Mamtimin 2011 (67)	N	Y	N
Mathé 2014 (68)	N	N	Y
McCullough 2021 (69)	N	N	N
Meller 2016 (70)	N	N	N
Men 2020 (71)	N	Y	N
Mondul 2015 (72)	N	N	N
Moore 2018 (73)	N	N	N
Moore 2021 (74)	N	N	N
Ose 2021 (75)	N	N	N
Petrick 2019 (76)	N	N	N
Piyarathna 2018 (77)	N	N	N
Röhnisch 2020 (78)	N	N	N
Ros-Mazurczyk 2017 (79)	N	N	N
Schmidt 2017 (80)	N	N	N
Schmidt 2020 (81)	N	N	N
Seow 2019 (82)	N	Y	N
Shu 2018 (83)	N	N	N
Shu 2018 (84)	N	N	N
Stepien 2021 (85)	N	N	N
Stolzenberg-Solomon 2020 (86)	N	N	Y
Su 2019 (87)	N	N	Y
Sun 2019 (88)	N	Y	N
Vanhove 2018 (89)	N	Y	Y
Wang 2016 (90)	N	Y	N
Wang 2021 (91)	N	N	N
Wei 2021 (92)	N	N	N
Wikoff 2015 (93)	N	N	Y
Wilson 2013 (94)	N	N	N
Yamakawa 2017 (95)	N	N	N
Yi 2014 (96)	Y	Y	N
Zeleznik 2020 (97)	N	N	N
Zhang 2020 (98)	N	Y	Y
Zhao 2019 (99)	N	Y	Y

[a]These data were extracted by one author (KAZ) and 10% of these data were checked for quality control by a second author (EL).
[b]Represents discrimination statistics other than receiver operating characteristic curves.
[c]Validation set includes both external validation and internal replication.
